# Aging in Language Dynamics

**DOI:** 10.1371/journal.pone.0016677

**Published:** 2011-02-17

**Authors:** Animesh Mukherjee, Francesca Tria, Andrea Baronchelli, Andrea Puglisi, Vittorio Loreto

**Affiliations:** 1 Institute for Scientific Interchange (ISI), Torino, Italy; 2 Departament de Fisica i Enginyeria Nuclear, Universitat Politecnica de Catalunya, Barcelona, Spain; 3 CNR-ISC, Roma, Italy; 4 Dipartimento di Fisica, “Sapienza” Università di Roma, Roma, Italy; University of Maribor, Slovenia

## Abstract

Human languages evolve continuously, and a puzzling problem is how to reconcile the apparent robustness of most of the deep linguistic structures we use with the evidence that they undergo possibly slow, yet ceaseless, changes. Is the state in which we observe languages today closer to what would be a dynamical attractor with statistically stationary properties or rather closer to a non-steady state slowly evolving in time? Here we address this question in the framework of the emergence of shared linguistic categories in a population of individuals interacting through language games. The observed emerging asymptotic categorization, which has been previously tested - with success - against experimental data from human languages, corresponds to a metastable state where global shifts are always possible but progressively more unlikely and the response properties depend on the age of the system. This aging mechanism exhibits striking quantitative analogies to what is observed in the statistical mechanics of glassy systems. We argue that this can be a general scenario in language dynamics where shared linguistic conventions would not emerge as attractors, but rather as metastable states.

## Introduction

A wide open question about the emergence and the evolution of shared linguistic conventions concerns the role of timescales [1], [2]. Phonetic, morphological, semantic, syntactic features of language vary over time. A fair degree of variability of words and grammatical structures can be observed in the diachronic study of a given language [3], and the proportions of different linguistic variants used by individuals within a population are not constant, but rather shift within time [4]. An interesting case study is offered by linguistic categories, the classical and prototypical example being that of basic color terms [5]–[8], or simply “colors”. For example, from Old to Middle English the meaning of color terms shifted progressively from a brightness meaning sense to the present-day hue sense [9], and similar shifts have been documented for a wide array of languages [10]. Also the variability existing across different languages is an evidence of the continuous change of linguistic categories, as pointed out by the data gathered in the World Color Survey (WCS) [5], [11], [12]. At the same time, statistical analysis over a large number of languages have shown that different color naming schemes share universal patterns [13], and, after a long debate, the existence of universalities is nowadays widely accepted [6]–[8].

General principles of categorization [14] have been claimed to be sufficient to account for the observed universality [15]. For example, it has been suggested [16] that the simple principle according to which categories are constructed as to maximize similarity within categories and to minimize it across categories could be responsible for cross-linguistic similarity, and a quantitative analysis based on this intuition has confirmed its validity [17]. Alternatively, it has been hypothesized that weak perceptual biases could do the job [18]–[20], and recent numerical simulations have shown that this might well be the case [21]. In any case, both hypotheses have to deal with the existence of a large variability, which is reflected in the acknowledgment of the existence of non-optimal categorizations [17] and in the consideration of the weakness of the advocated perceptual biases [21]. But if optimality is the leading principle, how can languages get stuck in suboptimal categorization schemes? Or what does it mean that perceptual biases are “weak”? i.e., why are they not able to drive the evolution towards the very same end? And, more in general, which is the mechanism that allow languages to appear static while they are yet evolving?

Here we focus on the emergence of shared categorization patterns in the framework of the so-called Category Game (CG) [22], a language game [23] through which a population of individuals establishes a shared categorization that quantitatively reproduces the average correlation among different human languages as measured in the World Color Survey experiment [21]. A detailed analysis of the CG dynamics reveals that the physics of glassy systems [24], [25] can be the proper framework to formalize the intuition that languages change at the same time because of and notwithstanding the fact that they are the outcome of a collective behavior [26]–[28]. Languages are described as metastable states of global agreement, reconciling the evidence that they do continuously evolve [29]–[33] and they are at the same time stable enough to be intelligible across a population.

The physics of a so-called glass-forming liquid is such that when rapidly undercooled under its melting temperature, it looses it ability to flow on experimental time-scales and freezes in an amorphous state with huge rheological times, while the most stable state - the crystal - is never reached [24], [25]. This slowing down process can be quantified through the so-called relaxation time which turns out to be proportional to the viscosity of the fluid. Despite languages and glassy systems stand apparently very far apart, it is very intriguing to explore the analogy between a linguistic system and a dynamical system featuring glassy properties.

Within this perspective, we study the dynamics of the CG model with the tools of glass theory. In particular, we focus on the main aspects which are peculiar to these physical systems, i.e., the scaling of relaxation times and correlation functions with the population size as well as with the age of the system. In particular, the larger is the time over which one observes the system, formally known as the waiting time, the slower will be its response, i.e., its ability to undergo large-scale changes. From this perspective, the Category Game exhibits a glassy behavior and constitutes a first quantitative evidence of a very interesting link between cognitive science and the physics of glassy systems.

## Results

**Figure 1:**
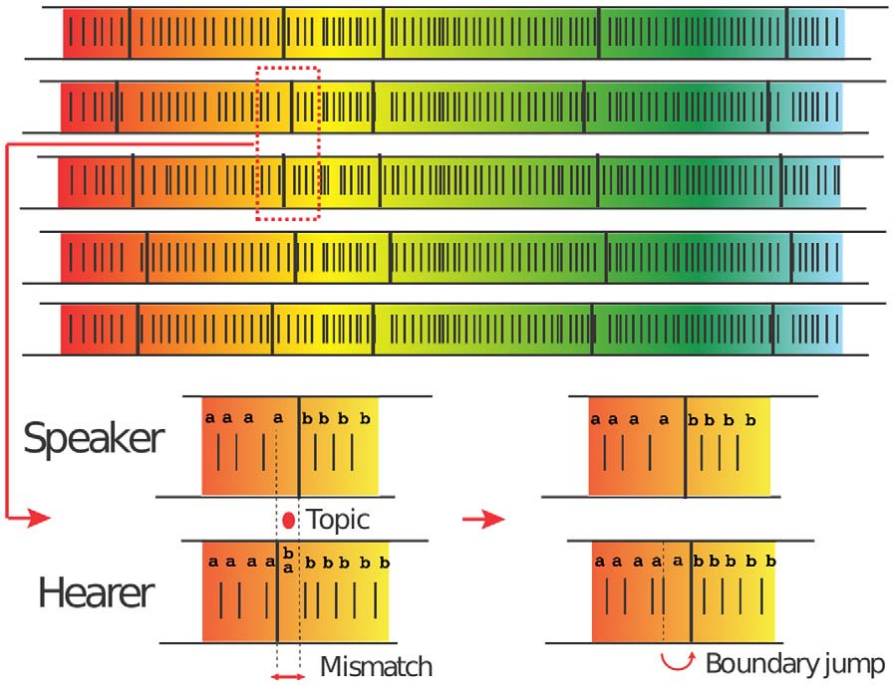
Typical long-time configuration of five representative agents in the population. For each agent perceptual and linguistic categories (separated by short and long bars, respectively) are shown. The highlighted portion of two agents illustrates an instance of a successful game in a so-called mismatch region between the linguistic categories of the two agents associated with the words “a” and “b” (see Materials and Methods for details). The hearer - in a previous game - learned the word “a” as a synonym for the perceptual category at the leftmost boundary of the linguistic category “b”. During the game the speaker utters “a” for the topic; as a result the hearer deletes “b” from her inventory, keeping “a” as the name for that perceptual category, moving *de facto* the linguistic boundary.

The Category Game [22] (see Materials and Methods for the details) describes the emergence of a hierarchical category structure made of two distinct levels: a basic layer, responsible for fine discrimination of the environment (perceptual categories), and a shared linguistic layer that groups together perceptions to guarantee communicative success (linguistic categories). At each time step a pair of individuals (one will be denoted as the speaker and the other as the hearer) is randomly selected from the population to play a language game that allow them to co-evolve the structure of their categories as well as their form-meaning inventories. Fig. 1 depicts a typical long-time configuration of the emerging category structure. While the number of perceptual categories (separated by short bars in fig. 1) is tuned by a parameter of the model (see Materials and Methods) and can be arbitrarily large, the number of linguistic categories (separated by long bars in fig. 1 and grouping together several perceptual categories sharing the same word) turns out to be finite and small, as observed in natural languages (for instance, like the basic color names across languages).

In the following, we report three sets of measures to establish the emergence of aging in the Category Game.

### Persistence of the Linguistic Categories

**Figure 2:**
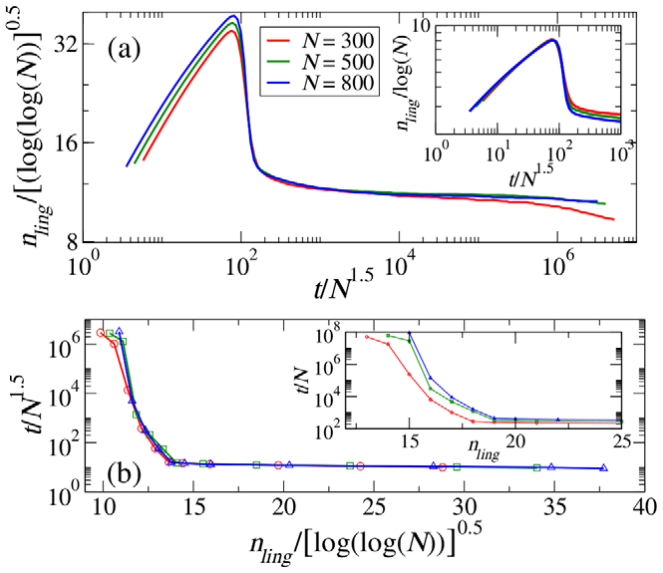
Persistence of the linguistic categories. (a) The rescaled average number of linguistic categories 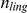 versus the rescaled number of games for three different population sizes (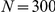, 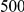 and 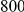). The plateau behaviour for the average number of linguistic categories is collapsed by rescaling the ordinate by 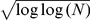 and the abscissa by 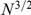. The inset shows the data collapse for the first part of the evolution where the ordinate is rescaled by 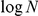 and the abscissa by 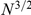. (b) The rescaled persistence time of 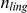 (i.e., the time spent by the system in a configuration corresponding to an average of 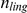 linguistic categories) versus the rescaled 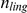 for 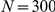, 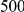 and 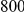 (legends correspond to those in (a) except that the curves are plotted with both lines and symbols here). Once again the ordinate is rescaled by 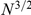 and the abscissa by 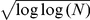 for data collapse. The inset shows a zoomed and uncollapsed version of the data (indicating the need for the collapse). Here the value of 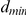 is set to the average human JND 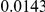 [38].

We start by investigating the dynamics of the number of linguistic categories emerging in the repertoire of each individual in a population. Two regimes are clearly distinguished (fig. 2a): initially, corresponding to a series of uncorrelated games, the average number of linguistic categories per individual 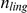 exhibits a rapid growth due to the pressure of discrimination (for a detailed description of CG we refer to the Materials and Methods section), followed by a rapid drop due to the onset of consensus and the merging of perceptual categories. A second regime is characterized by a quasi-arrested dynamics signaled by a “plateau” region, corresponding to a value of the average number of linguistic categories of the order of ten [21], [22]. Interestingly, the dependence of the number of linguistic categories on the population size 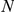 is different in the two regimes. In the first one, the average number of linguistic categories scales with 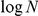 (see inset of fig. 2a), while in the second regime the dependence of the height of the plateau on the population size is extremely weak (O(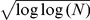)): the average number of linguistic categories in the population remains limited to a small value (of the order of 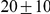) even for very large population sizes (up to billions of individuals). Furthermore, in the first regime we recover a time dependence on the population size of order 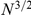 (fig. 2a), with a similar behaviour as in the Naming Game [34], [35], while the length of the plateau features a much stronger dependence on 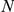, reaching a practically infinite value for large populations. At very large times, when the population is finite, the average number of linguistic categories starts to drop. We shall come back to this finite-size effect later on in the article. Most importantly, at the onset of the plateau region we observe a slowing down of the dynamics signaled by the divergence of the persistence time (fig. 2b). The plateau region is thus the interesting regime describing the persistence and evolution of the category system, and we will next describe its properties by looking at a more sophisticated dynamical quantity.

### Autocorrelation function and metastability

**Figure 3:**
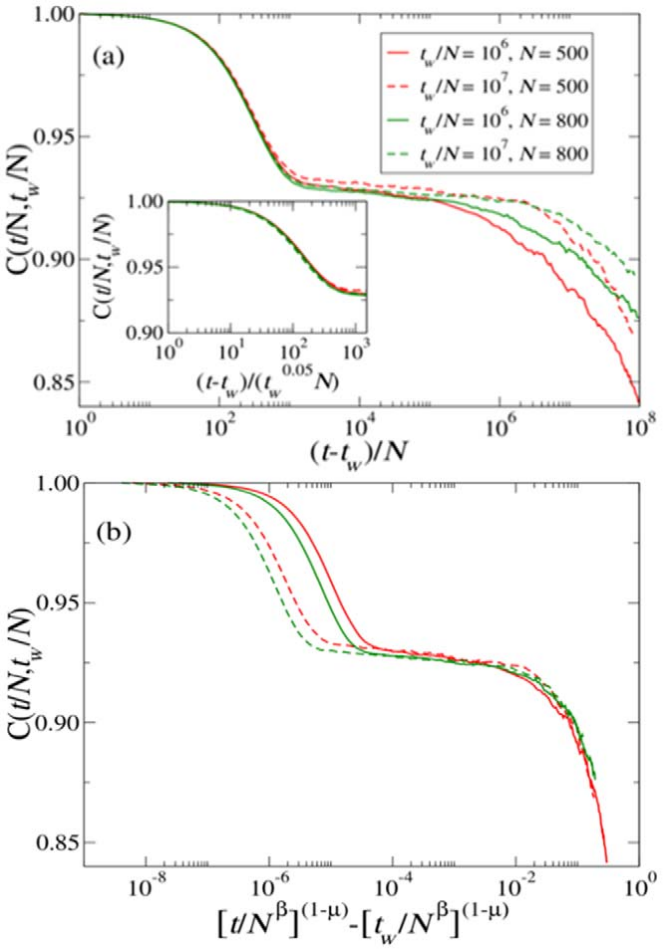
Relaxation of the correlation functions. (a) The autocorrelation 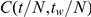 for 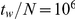, 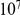 and 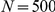, 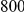. The inset shows the collapse of the 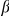 relaxation regime. In this regime, there is a very weak violation of the dependence of 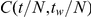 on 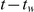 (time-translation invariance). (b) The collapse of the autocorrelation functions shown in (a) in the 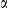 relaxation regime indicating sub-aging (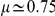). This result shows that the relaxation is strongly dependent on the size of the population (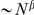 with 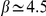). Here again the value of 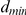 is set to the average human JND 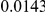 [38].

A system is said to be in dynamical equilibrium when it shows invariance under time translations; if this holds, any observable comparing the system at time 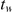 with the system at time 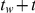 does not depend on 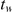. In contrast, a system undergoing aging is not invariant under time translation, i.e., time is not homogeneous. This property can be revealed by measuring correlations of the system at different times. Here we consider a suitably defined autocorrelation function, which we term 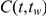: at time 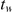 we save a copy of the configuration of all the agents in the population and subsequently, at time instances greater than 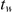, we compute the linguistic overlap of each agent with its copy saved at 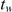; finally, we average this quantity over all agents (see Materials and Methods for detailed definitions). Results are presented in fig. 3 for two different population sizes. We recognize two different time scales, which we can associate to local or individual (fast) and collective or population-related (slow) dynamics. In particular, for 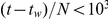, 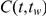 depends (almost) only on 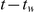 (see inset of fig. 3a). This phenomenon corresponds to what is known in the physics of glassy systems as the 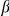-relaxation regime. This fast dynamics corresponds to the microscopic dynamics of the boundaries between linguistic categories at the individual level.

On the other hand, for 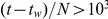, 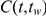 reveals aging, corresponding to the so-called 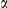-relaxation regime in glassy systems. This slow dynamics corresponds to the collective dynamics of the boundaries between linguistic categories at the population level. We find, in particular, for a fixed population size, a dependence on 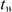 of the form:
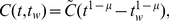
(1)with 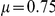 (see fig. 3b). Note that the same type of dependence on 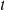 and 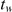 is also found in correlation functions of real glasses [36], [37], making quantitative the analogy of the CG dynamics with the dynamics of real out-of-equilibrium physical systems that exhibit sub-aging behavior. Let us simply recall that in the limit 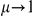 one recovers a pure aging behaviour since 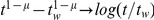. In the opposite limit, 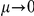, one recovers invariance under time translations.

Fig. 3 also reveals that the dependence of the length of the plateau on the population size is of the order of 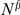 with 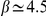. This suggests that the attractor of the dynamics, where a single linguistic category spreads over the whole interval, is practically never reached for large enough population sizes, and metastable states with a limited number of linguistic categories last for a practically infinite time.

### Finite-size effects

**Figure 4:**
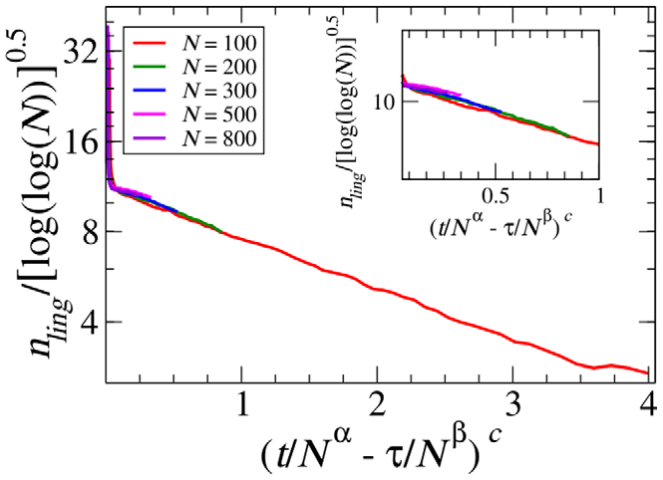
Finite-size effects. The rescaled average number of linguistic categories 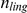 versus the rescaled number of games for five different population sizes (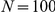, 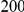, 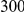, 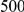 and 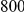). The bending region of the curves is collapsed by rescaling the number of linguistic categories by 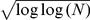 and the time axis as 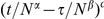 where 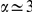, 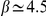, 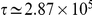 and 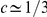. The inset shows a zoomed version of the same plot to present a better visualization of the data collapse.

In this section, we consider finite-size effects. We focus, in particular, on the behaviour of the average number of linguistic categories as a function of time (as observed in fig. 2a). A careful observation reveals that for very long times the plateau behaviour leaves room for a bending of the curves leading to a reduction in the average number of linguistic categories. This bending occurs earlier for small populations, i.e., for small system sizes. Fig. 4 shows the collapse of the curves for the average number of linguistic categories for different system sizes from 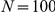, for which the bending is stronger, to 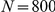. The collapse is aimed at superimposing only the bending region. It turns out that one collapses the curves after a rescaling of the abscissa as 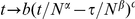, where the term 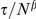 allows to superimpose the onset of the bending region while the term 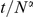 is the time rescaling well inside the bending region and 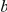 is a constant. The value of 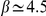 is consistent with what is observed in the collapse of the correlation function (see fig. 3) and confirms the idea that the length of the plateau region is scaling with a large power of the system size. On the other hand the bending region exhibits a characteristic time scaling as 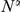 with 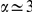 and the overall behaviour is well fitted by a stretched exponential function with an exponent 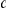 close to 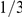.

**Figure 5:**
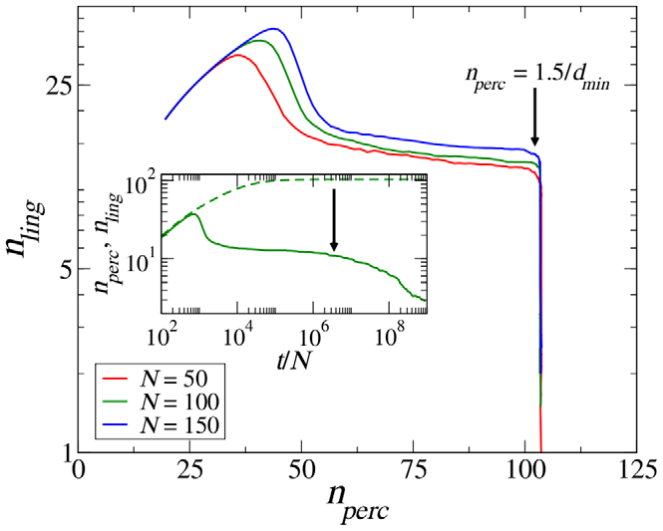
Linguistic vs. perceptual categories. Parametric plot of the number of linguistic categories vs. the number of perceptual categories, 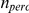, for different population sizes for which the bending region is accessible within a reasonable time (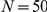, 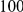 and 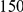). It is evident that there is a transition (indicated by the bold arrow) between a long-lasting regime where the number of perceptual categories keeps increasing, though at a very slow pace, and a regime where discrimination stops, the number of perceptual categories does not increase anymore and one observes only a decrease in the number of linguistic categories. The inset shows one representative example of the time evolution of 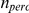 and 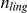 for 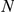 = 100 where the bold arrow marks the onset of the bending.

The onset of the bending region is marked by a clear phenomenon occurring in the structure of the perceptual and linguistic categories. In the plateau region discrimination keeps taking place, though at a slow pace, while at the onset of the bending region discrimination ceases and one is left with a pure dynamics of the boundaries between linguistic categories (see fig. 5). A detailed description of the dynamics of the domain boundaries in the plateau and in the bending region is out of the scope of the present paper and it will be presented elsewhere. It is nevertheless interesting to mention that one can describe the dynamics of the domain boundaries between linguistic categories in terms of correlated random walkers. The crucial difference between the plateau region (where aging occurs) and the bending region is that when the system ages the number of perceptual categories, which represent the underlying lattice where the random walkers can diffuse, is an increasing function of time while it is a constant when finite-size effects start.

## Discussion

In summary, our *in silico* experiment demonstrates a strong analogy between the slow dynamics of the Category Game and that of a suddenly quenched glass-former, in many different crucial aspects. The relaxation time of the number of linguistic categories shows a huge increase, similar to a singularity, at a finite number of categories: the system traps itself in a metastable state and the dynamics appears arrested even though the final state (only one category) is far from being reached. The dependence of the number of linguistic categories on the population size appears to be extremely weak (slower than a logarithm), and this could account for the universality of the number of color names (between 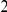 and 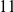) among different languages. The decorrelation of the system, apart from being slow, is also age-dependent, thus suggesting a possible explanation for the existence of more stable conserved properties of a language.

Taking a wider perspective, our results suggest that glassy behavior could provide important conceptual and technical tools to address the general problem of language change from a new perspective. Metastability, for example, allows to unfold the seemingly paradoxical nature of language change, according to which languages evolve because they are spoken by a large number of speakers but the evolution is frustrated since the speakers are indeed many. These intuitions are of course not a novelty (see for instance [32], [33]), but for the first time they have been properly quantified, in a numerical model closely connected to experimental data, and addressed in a well established framework. Future work could take into account crucial phenomena like language contact or more general cultural evolution processes, thus paving the way for a comparison with true historical data.

## Materials and Methods

### The Category Game

The computational model used for this study, introduced in [22], investigates how a population of individuals can develop a shared repertoire of linguistic categories, i.e., co-evolve their own system of symbols and meanings, without any pre-defined categorization and by only means of elementary language games [23]. A population of 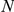 artificial agents is considered and to each agent (or individual) a continuous perceptual space (e.g., the visible light spectrum) is associated which, without any loss of generality, is assumed to be the interval 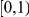. A categorization pattern refers to a partition of this interval into sub-intervals, or perceptual categories. Each individual has a dynamical form-meaning repertoire linking perceptual categories (meanings) to words (forms) representing their linguistic counterpart. The perceptual categories and the words associated with them co-evolve through a sequence of elementary language games among the agents. Initially, all individuals have a single perceptual category 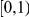 and no name associated to it.

At each time step a pair of individuals (one will be denoted as the speaker and the other as the hearer) is randomly selected from the population and based on the success or failure of communication, both rearrange their form-meaning inventories. Both the speaker and the hearer are presented with a scene made of 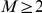 stimuli (objects), where a stimulus is a real number in the interval 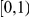. Without any loss of generality we will use in all the simulations 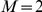. Any two objects in the scene cannot appear at a distance closer than 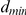: this is the only parameter of the model, fixing a minimal length scale which encodes a non infinite resolving power of any perception, for instance, the human Just Noticeable Difference (see below) in the case of colors. One of the objects is randomly selected to be the *topic* of the game and is known only to the speaker. The speaker checks whether the topic is the unique stimulus corresponding to one of its perceptual categories. If the two stimuli fall in one perceptual category, then the category is divided into two new categories by a barrier located in the center of the segment connecting the two stimuli. Both the new categories inherit the words associated to the original category plus a new word; this process is termed as *discrimination*. Subsequently, the speaker utters the most relevant name of the category containing the topic, where the most relevant name corresponds to either the last name used in a winning game or the new name in case the category has just been created. If the hearer does not have a category with this name, the game is a failure. If the hearer recognizes the name and has any object in one or more categories associated with that name, then it picks randomly one of these objects. If the object picked is the topic, then the game is a success; otherwise, it is a failure. In case of failure, the hearer learns the name used by the speaker for the category corresponding to the topic. In case of success, that name becomes the most relevant for that category and all other competing names are removed from the inventory associated with the category for both the players. An example of the evolving dynamics is shown in fig. 1.

It is worth to remark that in the framework of the CG model it was recently possible to reproduce the outcomes of the World Color Survey (WCS) [21]. This is a first evidence that the model can account for the *universality* of categorization patterns across cultures by means of only weak constraints on the perceptual space of the individuals. In [21], universal categorization patterns have been identified among populations whose individuals are endowed with the human Just Noticeable Difference (JND) function, describing the resolution power of the human eye to variations in the wavelength of the incident light [38]. In the simulations presented here, for the sake of simplicity, we adopt a constant 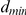 equal to the average human JND (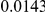), after having checked that the dynamical properties do not depend on eventual modulations of the JND function.

### A fast implementation of the Category Game

In this paper, for the investigation of the long-time dynamics of the model we devised and adopted a fast version of the Category Game that we briefly describe here, referring to a forthcoming paper for further details. The central idea behind the fast algorithm we implement is that of avoiding all the unnecessary games without outcomes (i.e., without changes in the configuration of either the speaker or the hearer or both) by forcing that each two players' game has an outcome and rescaling time accordingly. In this way, we do not alter the original dynamics since we conserve the playing order of the pairs speaker-hearer as well as the probability of playing in a given region.

More concretely, at each step we extract one pair speaker-hearer according to the probability 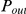 that an outcome will result if the pair plays, where an outcome is defined as any change in the repertoire of the speaker and/or the hearer. In the considered dynamics, we found that the probabilities 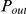 of all the pairs, at each given time, followed a peaked distribution, so that we could ultimately randomly extract one pair at each step without altering the results and significantly reducing the computational complexity. Furthermore, the region for placing the topic is extracted according to the probability that this choice will produce an outcome, and the object is extracted consistently. After each game time is increased by 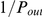. In this way time becomes a dependent variable. We checked that this fast implementation of the Category Game features the same dynamical properties of the original model for all the quantities of interest.

### Dynamical properties of the Category Game

**Figure 6:**
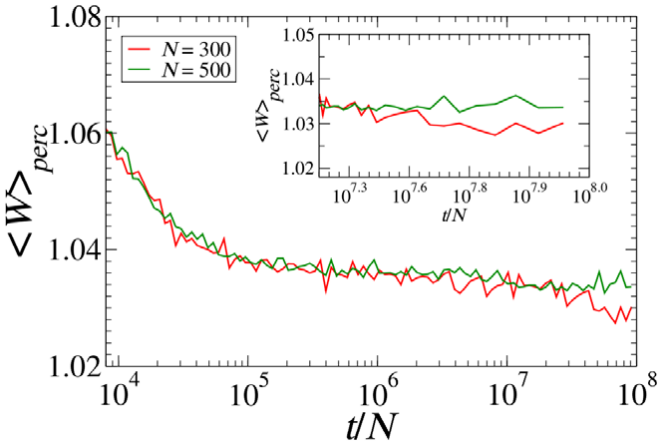
Words per perceptual category. The average number of words per perceptual category 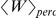 across the population of 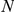 = 300, 500 agents versus the number of games per player. The inset is a zoom showing 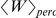 after 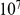 games per player. Clearly, 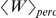 does not settle to one even after a very long time. The value of 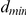 here is equal to 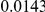 which is the average of human JND (when projected on the 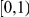 interval) [38].

In the Category Game dynamics it is possible to distinguish two different phases. In the first regime, the number of perceptual categories increases due to the pressure of discrimination, and at the same time many different words are used by different agents for naming similar perceptual categories. This kind of synonymy reaches a peak and then drops [22] in a fashion similar to the well-known Naming Game [34], [35], [39]. A second phase starts when most of the perceptual categories are associated with only one word (see fig. 6). During this phase, words are found to expand their dominion across adjacent perceptual categories. In this way, sets of contiguous perceptual categories sharing the same words are formed, giving raise to what we define as *linguistic categories* (see fig. 1). An important outcome thus is the emergence of a hierarchical category structure made of two distinct levels: a basic layer, responsible for fine discrimination of the environment, and a shared linguistic layer that groups together perceptions to guarantee communicative success. Remarkably, the emergent number of linguistic categories in this phase turns out to be finite and small [22], as observed in natural languages, even in the limit of an infinitesimally small length scale 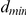, as opposed to the number of the underlying perceptual categories which is of order 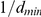.

### Linguistic overlap and autocorrelation function

The autocorrelation function 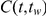 is defined as the average in the population of the individual linguistic autocorrelation, which, in turn, is defined as the overlap of the linguistic categories [22] of the considered individual at time 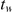, with itself at a later time 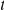. It then reads: 
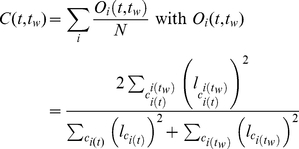
(2)where 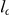 is the width of the linguistic category 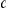, 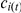 is a linguistic category of the 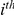 agent at time 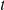 and 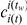 is the generic category of the intersection set containing all of the linguistic category boundaries of the agent 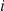 at time 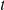 and its previous image saved at 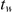. The function 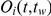 returns a value proportional to the degree of alignment of the two category inventories reaching its maximum unitary value when they are perfectly aligned.
